# MicroRNA-Based Diagnosis and Therapeutics for Vascular Cognitive Impairment and Dementia

**DOI:** 10.3389/fneur.2022.895316

**Published:** 2022-05-03

**Authors:** Weijie Zhai, Meng Zhao, Guimei Zhang, Zicheng Wang, Chunxiao Wei, Li Sun

**Affiliations:** Department of Neurology and Neuroscience Center, The First Hospital of Jilin University, Jilin University, Changchun, China

**Keywords:** apoptosis, blood-brain barrier, miRNAs, neuroinflammation, neurodifferentiation, oxidative stress, vascular cognitive impairment, dementia

## Abstract

Vascular cognitive impairment and dementia (VCID) is a neurodegenerative disease that is recognized as the second leading cause of dementia after Alzheimer's disease (AD). The underlying pathological mechanism of VCID include crebromicrovascular dysfunction, blood-brain barrier (BBB) disruption, neuroinflammation, capillary rarefaction, and microhemorrhages, etc. Despite the high incidence of VCID, no effective therapies are currently available for preventing or delaying its progression. Recently, pathophysiological microRNAs (miRNAs) in VCID have shown promise as novel diagnostic biomarkers and therapeutic targets. Studies have revealed that miRNAs can regulate the function of the BBB, affect apoptosis and oxidative stress (OS) in the central nervous system, and modulate neuroinflammation and neurodifferentiation. Thus, this review summarizes recent findings on VCID and miRNAs, focusing on their correlation and contribution to the development of VCID pathology.

## Introduction

Vascular cognitive impairment and dementia (VCID) is a term that ranges from mild cognitive impairment (MCI) to vascular dementia (VaD) and encompasses a continuum of cognitive disorders with cerebrovascular pathology (1). With an increase in the human lifespan, the number of individuals over the age of 60 in 2,050 is projected to be 2 billion (2), which will increase the demand for research on neurodegenerative diseases, especially those associated with aging. Age-related cerebrovascular factors are becoming recognized as a hallmark of VCID; thus, the number of patients affected by VCID is expected to exponentially increase in the upcoming decades (3) and be responsible for approximately 30% of the aging population living with dementia in Asia and developing countries (4).

VCID and dementia is a subtype of dementia affected by multiple factors, such as low educational attainment, female sex, stroke, and lifestyle (5). Current theories of the pathologies of VCID include cerebromicrovascular dysfunction, blood–brain barrier (BBB) disruption, neuroinflammation, capillary rarefaction, and microhemorrhages (3). However, none of the available pharmacological or preventative treatments decrease the development and progression of VCID. Improving our understanding of microRNAs (miRNAs) has provided strong epidemiological and experimental evidence that miRNAs are crucial for neuronal differentiation, survival, and activity (6). By inhibiting messenger RNA (mRNA) translation or promoting its degradation, miRNAs can regulate the expression levels of proteins and ultimately affect disease progression (7, 8). In the central nervous system (CNS), miRNAs can modulate the tight junctions of the BBB, affect the proliferation and differentiation of neuronal cells, and participate in the occurrence and development of neuroinflammation and oxidative stress (OS), resulting in neurodegenerative diseases and cognitive impairment and indicating that miRNAs have a more extensive influence on various brain diseases.

**Figure 1:**
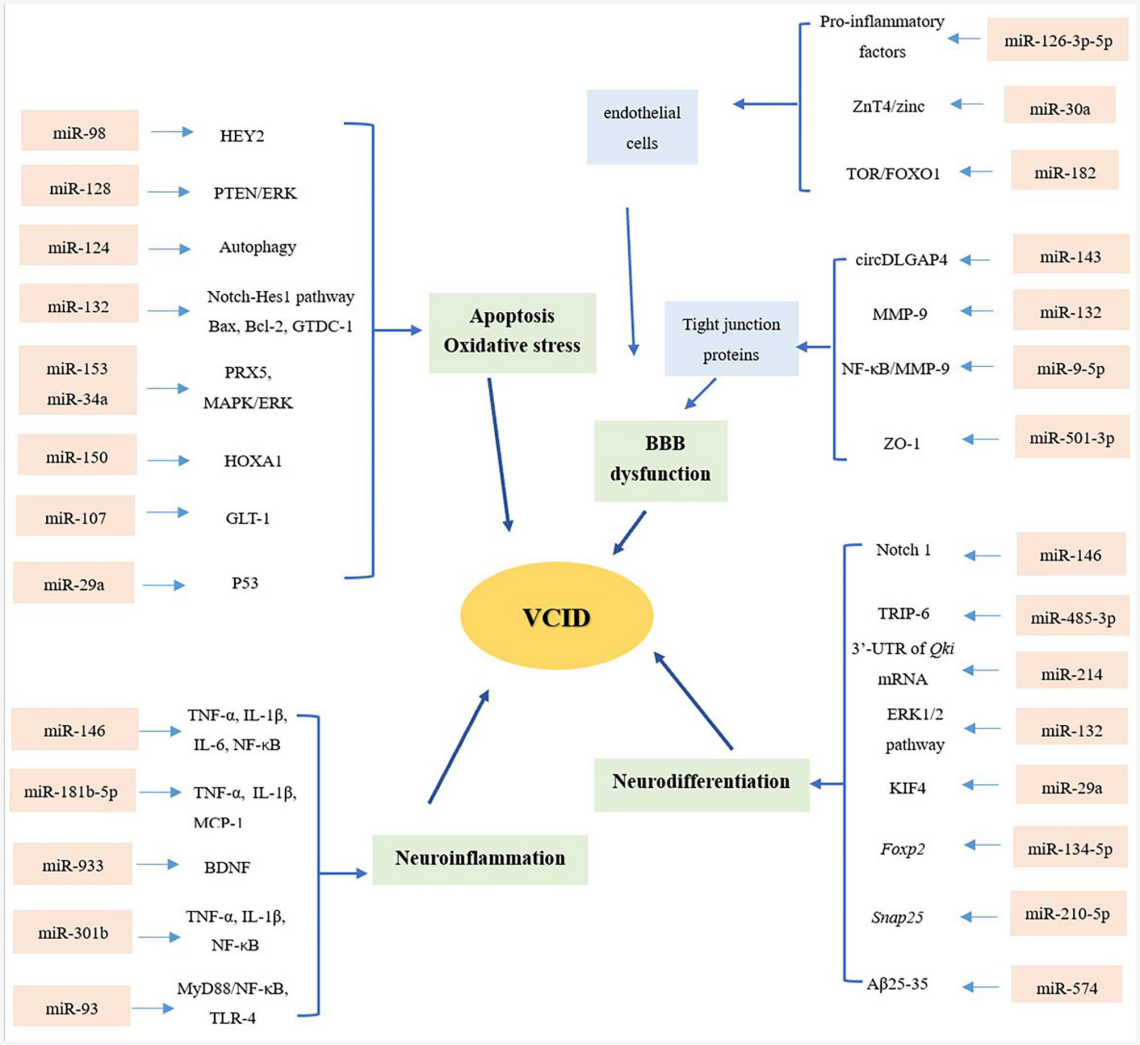
Overview of miRNAs and mechanisms of VCID. From the aspects of blood–brain barrier (BBB) dysfunction, apoptosis and oxidative stress, neuroinflammation and neurodifferentiation, microRNAs (miRNAs) playing multiple roles in the pathology of vascular cognitive impairment and dementia (VCID), providing a novel sight and promising targets in the diagnosis and treatment of VCID.

Biomarkers found in the CNS are typically present at relatively low concentrations in the BBB. The miRNAs are mainly transported within liposomes, high-density lipoproteins (HDLs), exosomes, and proteins that can protect them from degradation (9). Therefore, miRNAs are relatively stable compared to other biomarkers, and it is easy to monitor changes in their expression. Compared with Alzheimer's disease (AD), VCID is preventable and curable, suggesting that advanced diagnoses and reliable risk assessments will provide patients with more promising clinical outcomes. This review provides an up-to-date assessment of the role of miRNAs in VCID, from the series of depression of the BBB, apoptosis and OS, neuroinflammation, and neurodifferentiation (Figure 1).

## Development and Function of miRNAs

miRNAs are small non-coding RNAs of approximately 18–21 nucleotides that regulate gene expression by targeting mRNAs *via* cleavage and translational repression (10). The biogenesis of miRNAs starts with pri-miRNAs, which are transcribed by RNA polymerase II and converted into mature miRNA by RNase III enzymes such as Drosha and Dicer (11). Mature miRNAs leave the nucleus and are then combined with the Argonaute protein (AGO) or targeted mRNAs. It is understood that miRNAs can participate in and regulate molecular expression by interacting with 3′ untranslated regions (3′-UTRs) to post-transcriptionally affect the expression of target mRNA (11). Complete or partial function of miRNAs is also based on 3′-UTRs of target mRNAs (12). Several studies have shown that miRNAs may serve as novel therapeutic agents. While a single miRNA could target hundreds of mRNAs and influence the expression of genes involved in a functional interacting pathway (13), synergetic miRNAs share a common transcriptional regulatory mechanism on the levels of sequence, secondary structure, and transcriptional regulation (12). Depending on the construction of these miRNA–mRNA functional synergetic networks (MFSNs), the central role of these synergetic miRNAs can be analyzed in complex diseases (3). To further apply miRNAs in disease diagnosis and therapy, considerable research has been conducted to characterize their pathophysiological function.

The ability of miRNAs to target multiple genes within the signaling pathway makes them a promising target for modulation, and powerful regulators of cellular activities, such as cell differentiation, development, and apoptosis (14); synaptic homeostasis and plasticity processes (15); and angiogenesis in endothelial cells (ECs) (16) in CNS. In CNS, functional neurons are specialized and persistently renew the information required for constant neuronal adaptation to environmental clues (17), and the value of miRNAs has been recognized, especially for neural cells. As non-protein-coding molecules, miRNAs could modulate the function of tight junction proteins, ECs, astrocytes, and pericytes of BBB (18, 19), which help maintain the microenvironment of neural cells. To protect neural cells from apoptosis and OS, miRNAs can regulate the signaling pathway involved in modulating the proliferation or differentiation of cells (7, 20). Using public databases, such as the Gene Ontology (GO) annotation and Kyoto Encyclopedia of Genes and Genomes (KEGG) pathway enrichment analyses, the principles of miRNA–mRNA interactions in signaling pathways were further characterized and provide a novel insight into the mechanisms of disease progression (21).

## miRNAs and VCID

### miRNAs and BBB Dysfunction

As the most important protector of neurons in the brain, BBB exists between the blood microcirculation system and the brain parenchyma (19) to restrict the invasion of toxic substances, immune cells, and pathogens, playing an irreplaceable role in maintaining CNS homeostasis and proper function (22). Tight junctions constructed by ECs form the basic BBB structure, along with the basement membrane (BM), astrocytes, and pericytes, that contribute to the support and regulatory function of the BBB (23). However, the structural and functional integrity of the BBB is easily degraded by neuroinflammation, age-related degeneration, and other risk factors, which contribute to the onset and progression of cerebrovascular changes and neurodegenerative pathologies (24, 25).

Scientists have made great efforts to understand the molecular structure and pathological changes in the BBB, and have found that, compared with other cytokines, exosomes enriched with proteins and miRNAs can be easily released into the extracellular space, with functions ranging from blood coagulation to cell-to-cell communication (26). Ischemia is one of the most pathological processes of VCID that results in BBB dysfunction *via* inflammation or OS under the regulation of miRNAs and other factors (27, 28). A recent study showed that circular RNA DLGAP4 (circDLGAP4), which is an endogenous sponge of miR-143, *via* regulating the tight-junction protein and mesenchymal cell marker to inhibit the endothelial–mesenchymal transition, circDLGAP4 can significantly attenuate infarct areas and BBB damage, and is proposed to partially decrease the incidence of dementia (29). The miR-132 could target matrix metalloproteinase 9 (MMP-9) and dysregulate its expression, acting protection role in reducing the degradation of tight-junction proteins VE-cadherin and β-catenin to maintain the integrity of BBB (30). In addition, miR-9-5p can also mitigate BBB damage by activating the Hedgehog pathway and inhibiting the nuclear factor (NF)-κB/MMP-9 pathway (31). As mentioned earlier, ECs form the basic structure of the BBB. Of note, miR-126 participates in the maintenance of BBB integrity by regulating the functional status of ECs and attenuating BBB disruption by suppressing pro-inflammatory cytokines (32). It has been certificated that both *in vivo* and *in vitro*, miR-98 and miR-126-3p/-5p could significantly reduce the brain infract volume and improved behavioral outcomes (27, 32). Moreover, tumor necrosis factor alpha (TNF-α) modulates cerebral tight junctions and affects the BBB *via* the regulation of laudin-5 and tight-junction protein 1 (ZO-1) through the TNF-α-miR-501-3p–ZO-1 axis, resulting in working memory deficits and white matter lesions (33). In addition, it was found that overexpression of miR-Let7A does not only prevent brain endothelial (bEnd.3) cell death and inhibit pro-inflammatory responses but also protects tight-junction proteins from degradation under high-glucose conditions, indicating that miR-Let7A may be a novel solution for controlling BBB degradation, especially in patients with concomitant diabetes mellitus (DM) (34). Furthermore, exact mechanisms of how miRNAs affect the BBB integrity and ECs function have also been carried out, and miR-30a and miR-182 could modulate BBB permeability, tight-junction protein loss, and ECs apoptosis *via* ZnT4/zinc signaling pathway and mTOR/FOXO1 pathway, respectively (35, 36). Further study of complex physiological processes between miRNAs and BBB, and how to apply these mechanisms in clinic treatment are warranted.

### miRNAs and Apoptosis/Oxidative Stress

**Figure 2:**
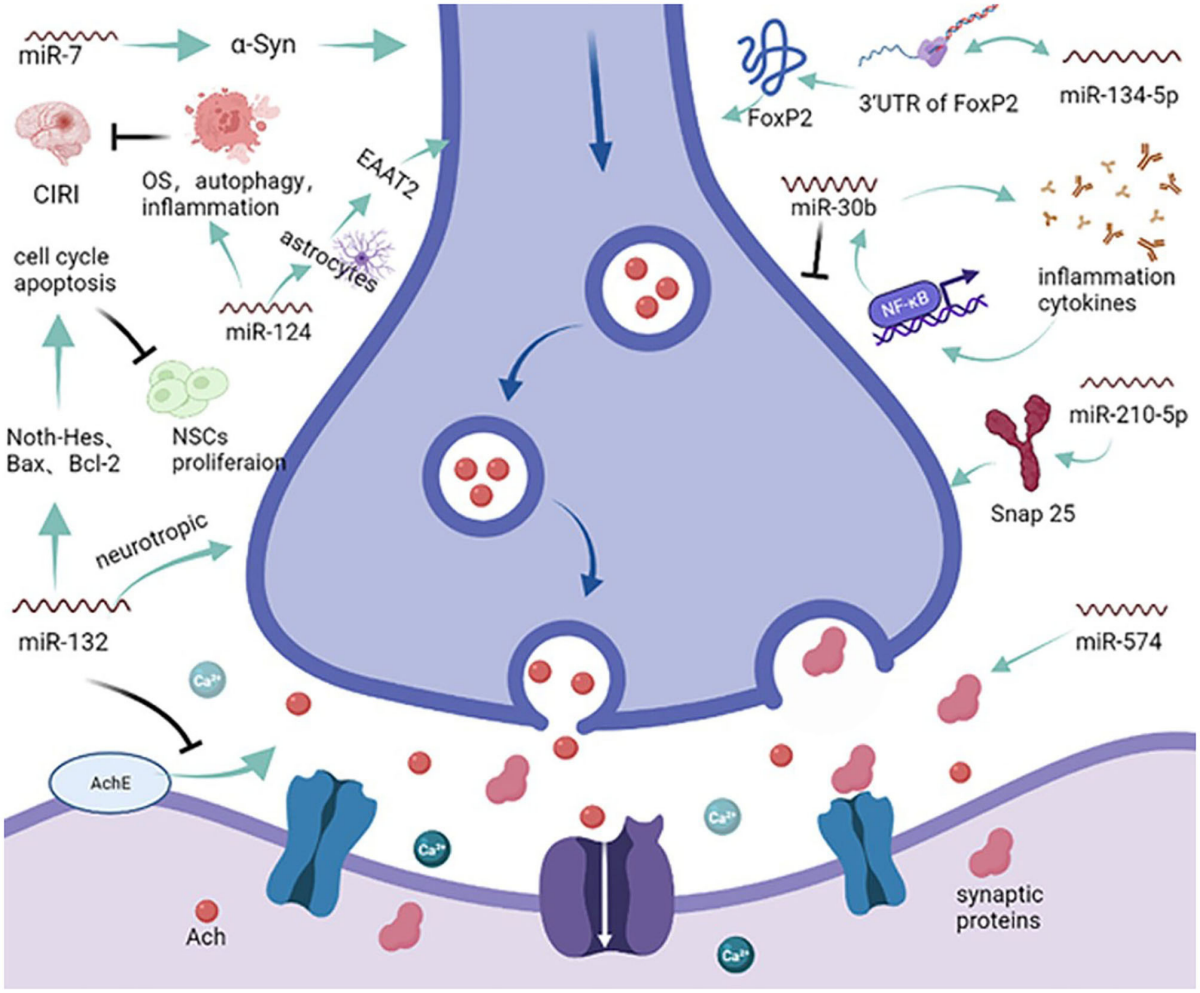
miRNAs and the synapse. MicroRNAs enveloped in exosomes can be released to target acetylcholine (Ach) and synaptic proteins, affecting the transmission of information at the synapse. miR-124 can modulate synaptic activation *via* the excitatory amino acid transporter 2 (EAAT2) and dysregulate oxidative stress autophagy to reduce cerebral ischemia reperfusion injury. Furthermore, miR-132 can modulate the degradation of Ach and dysregulate the proliferation of neural stem cells. On the other hand, miRNAs can also modulate synaptic neurodifferentiation *via* Snap 25, FxP2, and many other inflammatory cytokines.

Abnormal apoptosis is a prerequisite for endothelial and neuronal cell damage, resulting in the onset and progression of VCID (37). Additionally, synergistic and additive interactions between apoptosis and other signaling pathways add to the symptoms of VCID. Dementia caused by multiple infarcts implies that if strokes are prevented, so is the VCID that results from cerebral infarcts (38). Identifying pathways that predominantly include miRNAs during apoptosis will contribute to a better understanding of the functional overlap across diseases. As one of the most abundant miRNAs in brain tissue, miR-124a can be transported into astrocytes through neuronal exosomes, significantly increasing the expression of protein excitatory amino acid transporter 2 (EAAT2, rodent analog GLT1) and modulating synaptic activation (Figure 2) (39). However, the exact role of miR-124 has not been systematically elucidated. In age-related ischemic encephalopathy (IE), miR-124 can improve the effects of cerebral ischemic reperfusion injury (CIRI) by regulating OS, autophagy, and neuroinflammation but plays a negative role in synaptic plasticity and axonal growth *via* apoptosis (40). A greater understanding of miR-124 will open new avenues for further intervention in VCID. Increasing evidence suggests that cognitive decline in the early stages of neurodegenerative diseases, such as VCID, is a consequence of changes in synaptic structure and function (15). Based on analyses using online tools and a luciferase reporter assay, miR-132 was shown to protect acetylcholine (Ach) from degradation by most fast enzymes like acetylcholinesterase (ACHE) found in the body, and that miR-132 could also exert favorable effects on CNS neurons *via* brain-derived neurotrophic factors (BDNF) (41). In contrast, another report showed that miR-132 negatively regulates neural stem cell (NSC) proliferation by affecting the cell cycle and apoptosis through the Notch-Hes1 pathway, Bax, Bcl-2, and glycosyl transferase-like domain containing−1 (GTDC-1), which could induce neural cell apoptosis and tau phosphorylation (Figure 2) (20, 42). Due to the complexity of miRNAs, the key miRNA in these types of pathways and the construction of a shared-miRNA network is imperative. Previous studies have investigated the implications of α-synuclein (α-Syn), especially in inducing mitochondrial fragmentation, OS, and autophagy, which could promote neuronal cell death after stroke. It has been reported that treatment with miR-7 mimics greatly reduces the post-ischemic induction of α-Syn, significantly decreases the lesion volume, and improves motor and cognitive functional recovery (43). In addition, miR-153 and miR-34a have confirmed roles in protecting neural cells from death and apoptosis by upregulating peroxiredoxin 5 (PRX5) and MAPK/ERK signaling pathways, respectively, reducing the cell cycle and leading to a reduction in cell proliferation (44, 45), which could ultimately mitigate the pathology of dementia. Moreover, compared with the miR-150 mimic negative group, miR-150 overexpression significantly aggravated cell apoptosis by inhibiting the expression of homeobox (HOX) A1, which aggravates hippocampal neuronal apoptosis and cognitive impairment (46).

In addition to apoptosis, OS is also considered as one of the significant events in the pathological cascade of dementia, causing mitochondrial dysfunction and protein misfolding (47). Many studies in this field have shown that there is a mutual correlation between OS and miRNAs, and OS affects the expression of miRNAs, which have a counteracting effect on genes involved in OS (6). In the CNS, ECs play an irreplaceable role in protecting the cerebrovascular system from conditions, such as OS erosion, inflammation, and diabetes; however, OS has been predicted to predispose neurons to death in both direct and indirect ways (6, 48). Attenuating OS responsiveness could specifically limit the risk factors of cerebrovascular disease and improve endothelial homeostasis in vascular depression and VCID (49). The miR-107c and miR-29a could target glutamate transporter-1 (GLT-1) to modulate excitotoxicity in the CNS; miR-107c could target GLT-1 directly to evaluate the glutamate accumulation and neuronal excitotoxicity, whereas miR-29a mainly acts on p53 upregulated modulator of apoptosis (PUMA), which could attenuate OS and protect neurons from dementia caused by ischemic injury (50). The miR-128 is enriched in the CNS and easily detected in circulating lymphocytes and can inhibit neuronal damage caused by oxygen and glucose deprivation/reoxygenation (OGD/R) *via* the PTEN and ERK pathways (51). The correlation between miR-124 level and lesion size on CT indicated that miR-124 could be released from damaged brain tissue in patients who died within 3 months after suffering from a stroke (52). In contrast, by binding to the regulatory factor X1 (*RFX1*) mRNA, miR-124 could increase the expression of *RFX1*, resulting in the suppression of apolipoprotein E (APOE) and cellular amyloid beta (Aβ) in microglia, which could undermine the cognitive behavior of dementia (53). Another area of research in this field tracked the expression level of miRNAs in 45 patients with MCI and AD; notably, miR-146a and miR-181a were significantly upregulated in patients with MCI who later converted to AD, which was related to Aβ and APOE ε4 allele presence (54). This indicates that miRNAs can also be an indicator of disease progression, providing an insight in disease prediction. Furthermore, according to the measurement of OS-related proteins, superoxide dismutase and Na^+^, K^+^, and ATP, Chen et al. found that miR-98 could bind to the enhancer of split (Hes) related with the YRPW motif protein 2 (HEY2) to inhibit the production of Aβ and improve OS and mitochondrial dysfunction, providing a novel basis for targeted therapy for dementia (55).

### miRNAs and Neuroinflammation

Dynamic changes in miRNAs regulate the expression of genes involved in cognitive processes such as learning and executive abilities. Although the pathophysiology of VCID remains largely unknown, considerable efforts have been focused on neuroinflammation. Neuroinflammation is a hallmark of many neurological disorders, and pro-inflammatory or anti-inflammatory miRNAs within CNS signaling pathways can greatly aggravate or mitigate the pathological consequences of neurodegenerative diseases (56, 57). It has been reported that anti-inflammatory miRNAs (miR-21, miR-124, and miR-146a) and pro-inflammatory miRNAs (miR-27b, miR-155, and miR-326) regulate neuroinflammation by down or upregulating endogenous levels of immune receptors such as toll-like receptors (TLRs) or misfolded proteins that accumulate in the extracellular space (58). Related experiments conducted in BV-2 microglial cells and mice showed that miR-146 could suppress the release of pro-inflammatory factors [TNF-α, interleukin (IL)-1β, and IL-6] and the expression of mRNA in targeted cells; the upregulation of miR-146 could not only suppress the NF-κB pathway and microglial activation in the hippocampus but also promote hippocampus-dependent learning and memory capability (59). In addition to miR-146, mouse model and bioinformatics studies confirmed that miR-181b-5p can repress the expression of pro-inflammatory mediators such as TNF-α, IL-1β, and monocyte chemoattractant protein (MCP)-1. Injection of an miR-181b-5p mimic into the hippocampus of mice significantly improved cognition (60). Moreover, middle cerebral artery occlusion (MCAO)-induced behavioral disability and microglial activation in the brain were greatly improved and inhibited by miRNA-210-LNA (miR-210 inhibitor) post-treatment, providing new insight into the molecular basis of a novel therapeutic strategy (61). As the cholesterol metabolite, 27-hydroxycholesterol (27-OHC), induces discrete or directional inflammatory factors in microvascular endothelial cells (human microvascular endothelial cells, HMVECs) and increases the expression of miR-933 and inflammatory cytokines, which are elevated in plasma from dementia patients, more than that, *via* facilitates permeability and directional secretion from ECs into the brain, miR-933 may act as a paracrine inhibitor of neuronal BDNF, which provides a useful neuroprotective properties (62). These results show how miRNA functions in neuroinflammation and VCID progression, providing a novel insight on possible VCID interventions.

Before VCID occurs, a sustained pro-inflammatory environment brought about by NF-κB leads to chronic reactive astrogliosis, undermining the white matter (WM) (63). By interacting with NF erythroid 2-related factor 2 (Nrf2), NF-κB can fine-tune the cellular oxidative and inflammatory balance and participate in multiple pathologies in VCID, such as restoration of endothelial function and neurovascular coupling, reduction of amyloidopathy, and protection of WM integrity (64). To further implement miRNAs in disease mechanisms, counter intervention between miRNAs and NF-κB is ongoing. With advances in understanding that acupuncture can alleviate cognitive degeneration in VCID, in rats treated with acupuncture, TLR-4 was greatly dysregulated, accompanied by a decrease in miR-93 and MyD88/NF-κB signaling pathway activation (65). Furthermore, miR-301b accelerated cognitive impairment in mice with depression-like behavior; overexpression of miR-301b activated the NF-κB signaling pathway and aggravated inflammation in hippocampus, which accompanied the release of TNF-α, IL-1β, and many other cytokines (66).

### miRNAs and Neurodifferentiation

Vascular cognitive impairment and dementia typically refers to patients with both stroke and cognitive impairment. Recent studies have highlighted the roles of cerebrovascular injury, white matter tract integrity, microinfarcts, and secondary neurodegeneration in the development of VCID. The miRNAs modulate multiple biological functions, such as cell fate determination and differentiation. It has been found that the onset of cognitive impairment is accompanied by the senescence, loss, and neurogenesis decline of hippocampal neural stem cells (H-NSCs). Interestingly, embryonic stem cell-derived small extracellular vesicles (ESC-sEVs) can alleviate senescence and recover the compromised proliferation and differentiation capacity of H-NSCs *via* miR-17-5p, miR-18a-5p, miR-21-5p, and miR-29a-3p, which can inhibit the mammalian target of rapamycin complex 1 (mTORC1) activation (67).

**Figure 3:**
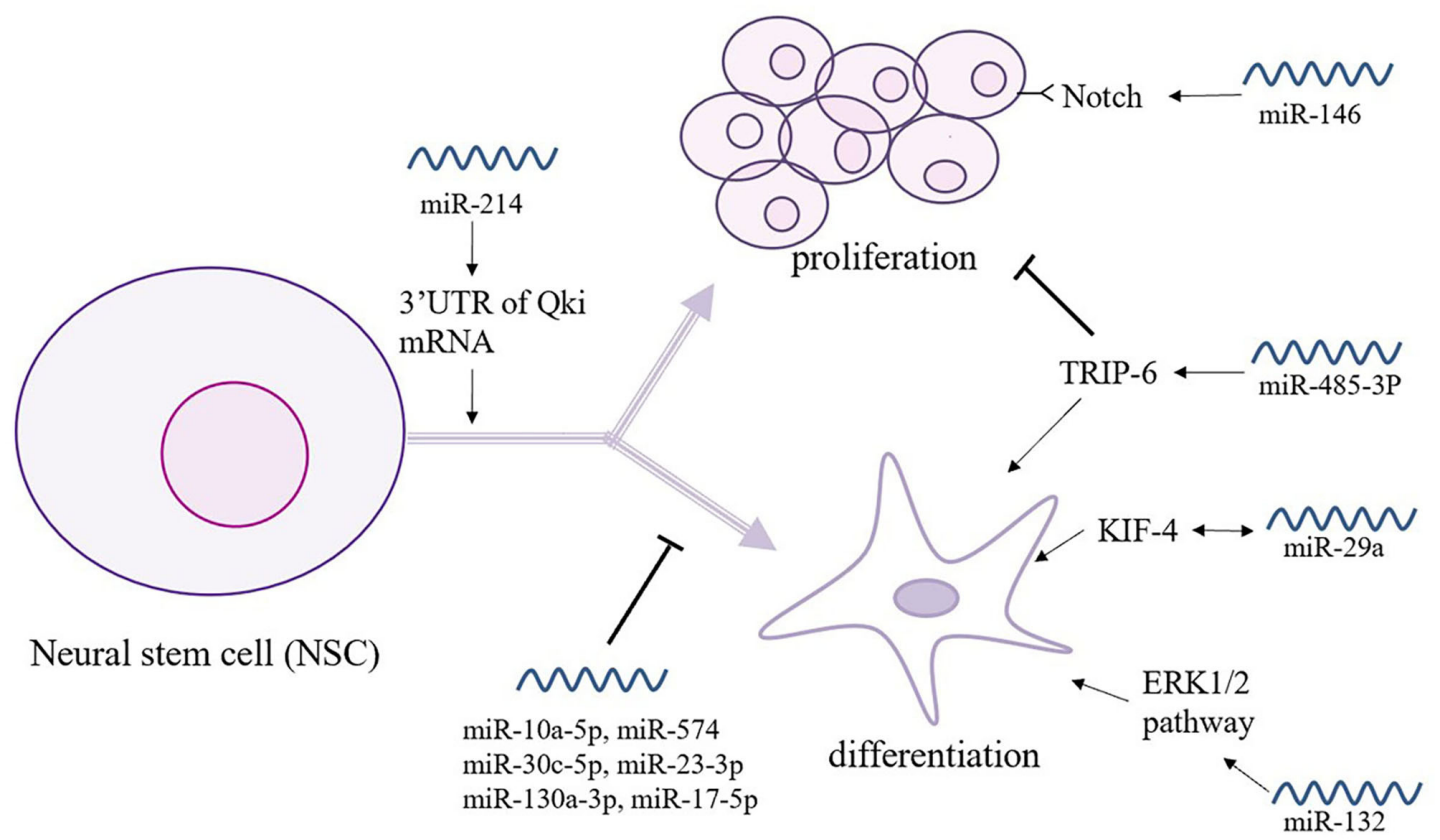
The relationship between miRNAs and neural stem cells (NSCs). NSCs play a key role in brain homeostasis and repair and show intrinsic pleiotropic properties. Thus, the intimate connections between NSCs and miRNAs are under study. As shown here, miR-146 and miR-585-3p play an opposite role in the proliferation and differentiation of NSCs. Moreover, a great number of miRNAs participates in the self-renewal and gene expression of NSCs.

Over the past 20 years, stem cell technology has become an increasingly helpful in the investigation and treatment of neurodegenerative diseases. NSCs participate in brain homeostasis and repair and show pleiotropic intrinsic properties, making them a promising candidate for the treatment of dementia (Figure 3) (68). Indeed, Notch signaling encodes a highly conserved cell-surface receptor that affects cell processes such as cell differentiation, cell apoptosis, and cell proliferation. In serum-free medium, miR-146 significantly promoted NSC proliferation by targeting the Notch 1 pathway but reduced the differentiation efficiency of glial cells (69). However, miR-485-3p has the complete opposite effect; miR-485-3p can reduce proliferation but can promote the differentiation of NSCs by decreasing TRIP-6 activity (70). Following the criteria and moderated *t*-statistics, miR-10a-5p was shown to attenuate the self-renewal of undifferentiated NSCs; however, similar to miR-574, miR-30c-5p, miR23-3p, miR130a-3p, and miR-17-5p miRNA families were predicted to decrease the expression of several genes associated with the differentiation of neurons, synapse formation, and neurite outgrowth (71). The miR-214 not only affects the differentiation of NSCs but also plays a key role that helps maintain the balance between proliferation and differentiation by binding the 3′-UTR of *Qki* mRNA, affecting downstream its signal transmission (72). Additionally, miR-132 is significantly overexpressed in differentiating NSCs and is accompanied by the activation of the ERK1/2 pathway, and it promotes glial cell differentiation *via* Mecp2 expression (20). During the process of neurodifferentiation, the level of miR-29a in plasma shows a time-dependent increase similar to that of Kruppel-like factor 4 (KlF4), which suggests that the regulation of miR-29a occurs through the KlF4 signaling pathway (73). This mechanism suggests that KIF4 may be used to promote miR-29a and provides a novel possibility for the treatment of VCID (73).

A widely accepted theory is that memory deficiency associated with cognitive impairment results from synaptic dysfunction. Another focus of research in this field is the investigation of the correlation between miRNAs and synaptic proteins (Figure 2). It has been found that miR-134-5p can act on Forkhead box P2 (*Foxp2*) mRNA to affect its level of expression; however, silencing *Foxp2* minimizes the effect of miR-134-5p on synaptic protein loss, which may prevent the development of cognitive impairment, especially in vocal learning (74). In addition, by comparing the expression level of miR-30b in APP transgenic (TG) and wild-type (WT) mice, miR-30b was significantly upregulated in TG mice, causing synaptic and cognitive dysfunction (75). Indeed, miR-30b is triggered by pro-inflammatory cytokines through NF-κB signaling, suggesting a feedback loop in the process of dementia. More specifically, real-time reverse transcription polymerase chain reaction (RT-PCR) revealed that overexpression of miR-210-5p can decrease the number of synapses in primary hippocampal neurons by targeting synaptosomal-associated protein, 25 kDa (*Snap25*) (76). In experimental trials that used APP/PS1 mice and WT mice, miR-574 could lower neuritin and synaptic protein expression in primary hippocampal neurons *via* targeting Aβ25-35, resulting aggravation of cognitive dysfunction in APP/PS1 mice compared with WT mice (77).

## The Perspective of miRNAs in the Diagnosis and Treatment of VCID

As the second most common cause of cognitive impairment, patients with VCID will be up to 150 million in 2,050. VCID has drawn the attention of researchers because it is preventable and curable; however, some neuroprotective agents have been reported to attenuate but not cure symptoms. Risk factors of VCID including protective factors, such as higher education, occupation and social networks, and others, increase the risk of dementia (38). According to the National Institute for Neurological Disorders and Stroke-Association International pour la Recherché et l'Enseignement en Neurosciences (NINDS-AIREN), the basic features of VCID include (1) acute impairment of memory and at least two other cognitive domains, (2) neuroimaging evidence of cerebrovascular lesions, and (3) evidence for a temporal relationship between stroke and cognitive loss (78). In recent years, miRNAs are regarded as cost-effective and non-invasive biomarkers in disease diagnosis and therapy response monitoring (79). Additionally, miRNAs are easily detected in biofluids like plasma and cerebrospinal fluid (CSF) due to their unique biological characteristics (15), which can provide biological and clinical breakthroughs.

To gain a better understanding of how miRNAs could support both diagnosis and therapy, studies of clinical patients and biological experiments have been conducted. Validation studies revealed that four miRNAs (miR-409-3p, miR-502-3p, miR-486-5p, and miR-451a) are potentially valuable biomarkers for identifying VCID with relatively high sensitivity and specificity (80). Moreover, combined receiver operating characteristic curve analysis of seven miRNAs revealed an area under the curve (AUC) of 0.64 with a sensitivity of 55.5% and specificity of 65.7%, whereas plasma miR-409-3p, miR-502-3p, miR-486-5p, and miR-451a could differentiate patients with VCID from healthy controls (80). To improve the diagnosis and anti-diastole level of dementia, the value of miRNAs in differential diagnosis protocols was determined. Through the measurement of miRNA expression levels in MCI, VCID, AD, and Parkinson's disease with dementia (PDD), miR-1, miR-384, and miR-19b-3p were identified as good diagnostic biomarkers and provided a novel insight in disease prevention (81, 82). Another study comparing different miRNAs expressed in AD, MCI, and healthy controls found that miR-455-3p, miR-4668-5p, miR-3613-3p, and miR-4674 were upregulated, whereas miR-6722 was downregulated in AD and MCI compared with healthy controls (83). More research is warranted concerning the clinical consequences of VCID and expression changes of miRNAs, especially in disease diagnosis and prediction (Table 1).

**Table 1:** Changes in miRNAs expression level and its role in disease diagnostics.

**miRNAs**	**Source**	**Expression level**	**Diagnosis**	**Reference**
miR-409-3p	Plasma	Down	Discrimination diagnosis	(80)
miR-502-3p		Up		
miR-486-5p		Up		
miR-451a		Up		
miR-1	Exosome	Down	AD, VaD, or PDD	(81), (82)
miR-384		Up		
miR-19b-3p		Down		
miR-455-3p	Serum	Up	AD, MCI, or healthy	(83)
miR-4668-5p		Up		
miR-3613-3p		Up		
miR-4674		Up		
miR-126	Mouse serum	Down	Neuroinflammation, water channel and glymphatic dysfunction	(84)
miR-191	Mice	Up	Apoptosis and proliferation dysregulation	(85)

A large number of fundamental experiments have been conducted to gain a better understanding of their signaling pathways and the post-transcriptional mechanisms of miRNAs. Comparison of astrocytic and microglial activation, WM damage, water channels, and glymphatic dysfunction in mice with miR-126 deletion (miR-126 EC^−/−^), and control (miR-126 flox/flox), miR-126 EC^−/−^mice showed significantly decreased cerebral blood flow (CBF) and increased inflammation that was accompanied by poor performance and cognitive deficits (84). As a vasoconstrictor factor, endothelin-1 (ET-1) was increased in the plasma of patients after stroke and in the CSF of patients with VCID (86). An miR-125a inhibitor substantially upregulated the expression of ET-1, whereas miR-125a and the presence of the rs12976445 minor allele polymorphism downregulated the delivery of ET-1 to ECs (87). These results suggested that miR-125a, ET-1, and rs12976445 have a promising potential as pathological solutions for post-stroke dementia.

Apoptosis and proliferation have been implicated in many diseases, confirming the negative correlation between miRNAs and cognitive impairment. It is known that miR-191 can aggravate apoptosis and misregulate proliferation and migration; *in vivo* studies have shown that applying an miR-191 antagomir significantly attenuated infarction volume by mechanically targeting vascular endothelial zinc finger 1 (*VEZF1*) transcript (85). In addition, miR-196a and LRIG3 enhanced learning and memory by ameliorating injury to hippocampal neurons *via* the PI3K/Akt pathway (88). Although these studies represent only the tip of the iceberg, they provide a novel insight into the multiple miRNAs that can intervene in signaling pathway function and affect the outcomes of patients with different forms of dementia and cognitive impairment.

Various treatments and interventions have been reported to be effective for dementia; however, no therapeutic cures are currently available. According to previous reports, symptomatic and alternative therapies are effective; however, antipsychotic treatment is less satisfactory (89). For VCID caused by vascular factors that may be mixed with AD pathological changes, more attention should be focused on therapeutic agents for synaptic protection, anti-pathologic therapeutics, and effective management of vascular risk factors (90). However, acupuncture is a promising alternative therapy and may be an underlying TLR4 inhibitor for VCID (65). Preclinical experiments indicate the advantages of remote ischemic conditioning (RIC) for decreasing the recurrence of ischemic stroke, and repetitive treatments with RIC in patients for 6 months show satisfactory outcomes, especially in neuropsychological assessments (91). Currently, therapeutics based on miRNAs are mainly focused on miRNA mimics and inhibitors (antagomirs), respectively, to adjust the expression level of target genes (92). Mice with transient middle cerebral artery occlusion showed that pre- or post-ischemic treatment with an miR-7 mimic decreased lesion volume and improved motor and cognitive functional recovery (43). Biomarkers can aid in early diagnosis before the pathology occurs, limiting disease progression, and potentially indicating patient response to the treatment. Thus, refining the use of biomarkers will allow dementia treatment to enter the era of precision medicine (93).

## Conclusion

This is an exciting time for miRNAs research because of the recent advancement and identification of miRNA genes, their expression patterns, and their regulatory targets in diseases such as VCID, AD, and other neurodegenerative diseases. The mechanisms discussed in this review provide *prima facie* evidence for the mutual effects of miRNAs on the pathogenesis of VCID. A vast number of miRNAs play roles in the mutual regulation in genes and proteins, and it has been reported that high- or low-expression level of miRNAs can improve or impair the pathological progress of diseases. Hence, we have highlighted the roles of miRNAs in the modulation of VCID. However, the clear interventions of miRNAs and VCID are still poorly reported, and further studies in this area are expected to emphasize the pathways and mechanisms that could improve disease and help develop VCID therapies.

## Author Contributions

WZ and MZ organized the content of the entire manuscript, wrote the sections, and were responsible for the figures. GZ, ZW, CW, and LS contributed substantially to the conception and design of the work. All authors read and approved the final manuscript.

## Funding

This study was supported by the grant provided by the Major Chronic Disease Program of the Ministry of Science and Technology of China (No. 2018YFC1312301) and the General Program of the National Natural Science Foundation of China (No. 82071442), The First Hospital of Jilin University, China.

## Conflict of Interest

The authors declare that the research was conducted in the absence of any commercial or financial relationships that could be construed as a potential conflict of interest.

## Publisher's Note

All claims expressed in this article are solely those of the authors and do not necessarily represent those of their affiliated organizations, or those of the publisher, the editors and the reviewers. Any product that may be evaluated in this article, or claim that may be made by its manufacturer, is not guaranteed or endorsed by the publisher.
